# Degradation and Biocompatibility of AZ31 Magnesium Alloy Implants In Vitro and In Vivo: A Micro-Computed Tomography Study in Rats

**DOI:** 10.3390/ma13020473

**Published:** 2020-01-19

**Authors:** Naohiko Kawamura, Yuya Nakao, Rina Ishikawa, Dai Tsuchida, Masahiro Iijima

**Affiliations:** Division of Orthodontics and Dentofacial Orthopedics, Department of Oral Growth and Development, School of Dentistry, Health Sciences University of Hokkaido, Hokkaido 061-0293, Japan; y-nakao@hoku-iryo-u.ac.jp (Y.N.); r-ishikawa@hoku-iryo-u.ac.jp (R.I.); tsuchida_d@hoku-iryo-u.ac.jp (D.T.); iijima@hoku-iryo-u.ac.jp (M.I.)

**Keywords:** magnesium, magnesium alloy, implant, bioabsorbable material, micro-CT, BV/TV, BIC, orthodontic, miniscrew

## Abstract

In current orthodontic practice, miniscrew implants (MSIs) for anchorage and bone fixation plates (BFPs) for surgical orthodontic treatment are commonly used. MSIs and BFPs that are made of bioabsorbable material would avoid the need for removal surgery. We investigated the mechanical, degradation and osseointegration properties and the bone-implant interface strength of the AZ31 bioabsorbable magnesium alloy to assess its suitability for MSIs and BFPs. The mechanical properties of a Ti alloy (TiA), AZ31 Mg alloy (MgA), pure Mg and poly-L-lactic acid (PLA) were investigated using a nanoindentation test. Also, pH changes in the solution and degradation rates were determined using immersion tests. Three-dimensional, high-resolution, micro-computed tomography (CT) of implants in the rat femur was performed. Biomechanical push-out testing was conducted to calculate the maximum shear strength of the bone-implant interface. Scanning electron microscopy (SEM), histological analysis and an evaluation of systemic inflammation were performed. MgA has mechanical properties similar to those of bone, and is suitable for implants. The degradation rate of MgA was significantly lower than that of Mg. MgA achieved a significantly higher bone-implant bond strength than TiA. Micro-CT revealed no significant differences in bone density or bone-implant contact between TiA and MgA. In conclusion, the AZ31 Mg alloy is suitable for both MSIs and BFPs.

## 1. Introduction

In current orthodontic practice, miniscrew implants (MSIs) are used as skeletal anchorages for tooth movement [1]. The concept of skeletal anchorage was introduced to orthodontics in the 1980s, and by the 2000s, they had been accepted worldwide [2,3,4,5,6]. MSIs are versatile, minimally invasive, inexpensive and are accepted by patients [7,8,9,10].

Two types of implant are used therapeutically: (1) Those used semi-permanently to restore the shape and function of the defect site; e.g., artificial joints, artificial dental roots and artificial blood vessels, and (2) those placed temporarily; e.g., bone fixation materials, stents and surgical sutures [11]. The former is required to have high durability, but the latter is degraded and absorbed after the living tissue has been repaired. Because MSIS are placed when a fixed source is needed, and then removed when it is no longer needed, they are categorized into the second category of implants. In addition, bone fixation plates (BFPs) for orthognathic surgery are placed temporarily, as are bone fixation materials for fractures. If the BFP is left in situ after healing, the bone undergoes stress shielding, which leads to bone resorption and fracture.

At present, passivated and highly corrosion-resistant metallic materials—e.g., the Ti–6Al–4V alloy, pure Titanium (Ti), Co–Cr alloy and 316L stainless steel, are used for MSIs and BFPs [12,13]. Surgery to remove these materials imposes a physical and economic burden upon the patient; therefore, such surgery can be avoided if bioabsorbable MSIs and BFPs were used [13,14].

Bioabsorbable materials include polymeric materials such as poly-L-lactic acid (PLA) and polyethylene glycol, and bioceramics such as β-tricalcium phosphate and hydroxyapatite [15]. These have inferior strength and toughness compared to metallic materials, and so are unsuitable for applications involving high loads. Iron (Fe), Magnesium (Mg) and Zinc (Zn) are three absorbable metals [16]. Mg has been the subject of much basic and translational research. Mg readily dissolves in vivo as follows, because it has a low standard electrode potential [1], and is of low toxicity [14]:

Mg → Mg^2+^ + 2e^−^ (1)

2H_2_O + 2e^−^ → H_2_ + 2OH^−^ (2)

Furthermore, Mg promotes new bone formation [17,18,19], and its properties make it suitable for use in bone implants. Mg has a higher in vivo corrosion rate and lower strength for the anchorage of tooth movement and bone fixation than other absorbable metals—e.g., Fe and Zn. The high in vivo corrosion rate of Mg can be decreased by alloying and advanced metallurgical processes [20]. Mg alloys exhibit high strength and toughness, and are safely degraded, absorbed and dissipated in vivo [13,15,17]. Mg alloys are 3–16-fold stronger than biodegradable polymers such as PLA, they are bioabsorbable, and have excellent ductility [21,22]. In addition, Mg alloys have an elastic modulus (45 GPa) similar to that of human cortical bone (20–40 GPa), and markedly lower than those of the Ti-based materials used for MSIs and BFPs (110 GPa) [11]. Generally, the elastic modulus of metals for bone implants is significantly greater than that of bone, and so the stress is not distributed evenly between the two (i.e., stress shielding), thus hampering bone remodeling and promoting bone absorption [13]. However, because Mg alloys have an elastic modulus similar to that of cortical bone, the effect of stress shielding is small, and as such, these alloys are suitable for MSIs and BFPs [12]. Pure Mg has inadequate mechanical strength for the anchorage of tooth movement and bone fixation than other absorbable metals, and so Mg alloys which have been developed for industrial use, as well as new Mg alloys, have been applied in dentistry [11].

A period of 3–6 months is required for healing the surrounding bone after the implantation of an MSI or a BFP, which remain in situ for ≥1 year. Because the implant site is subject to various stresses, tight osseointegration is essential. Inflammation of the surrounding bone, the location of the MSI, the thickness of the cortical bone, skeletal characteristics and age, are linked to MSI failure [23,24,25,26] due to the loss of contact with the bone. Therefore, implant materials must have a high affinity (i.e., a high contact ratio) for bone. Previously, two-dimensional histological images were used to evaluate the bone–implant contact ratio [12,27,28]. Regarding research on magnesium, prior studies quantified bone formation via histomorphometry to assess the osseointegration characteristics of degradable Mg alloy implants and scaffolds [17,29]. However, the samples had to be examined in two-dimensional sections, resulting in considerable sample losses [30]. Furthermore, the testing procedure destroys the sample, preventing subsequent analysis, such as mechanical extrusion testing. Therefore, we evaluated osseointegration using micro-CT. Micro-CT is nondestructive, and yields three-dimensional data with a spatial resolution of several micrometers, facilitating the reconstruction of a two-dimensional image in any plane [31]. In addition, the evaluation of osseointegration via histological observation is influenced by the operator, and so errors can occur. Although the resolution of micro-CT is inferior to that of an optical microscope, it is superior in terms of objectivity and accuracy, because examiners can evaluate the presence of contact by extracting all of the implants and the bone under the same conditions. A prior study evaluated the osseointegration of a Mg alloy using microcomputed tomography (CT) [32]. Castellani et al. performed micro-CT at a spatial resolution (voxel size) of 20 μm, which may not be sufficient to assess osseointegration, given the resolution of optical microscopes (200 nm). Micro-CT technology is now used in medicine and industry, and its resolution has increased. 

In this study, the microstructure of the bone-implant interface was analyzed three-dimensionally using high-resolution microfocus X-ray CT (micro-CT) with a spatial resolution (voxel size) of 7.0 μm.

Previous studies of Mg alloys as bioabsorbable materials focused on corrosion rates and biocompatibility. Mechanical push-out testing is widely used to evaluate peri-implant bone in orthopedic studies. However, few studies on Mg alloys used this method. Furthermore, the strength of the bone-implant bond determined by biomechanical testing is a more sensitive predictor of implant stability than histomorphological analysis [33]. Thus, the biomechanical testing of the bone-implant interfacial strength is essential for the evaluation of new biomaterials for bone implants.

We compared the in vivo degradation properties, bone-implant interface strength, osseointegration properties and biocompatibility of an Mg alloy (MgA) with those of a Ti alloy (TiA), pure Mg and PLA to assess the suitability of the MgA for MSIs and BFPs.

## 2. Materials and Methods

### 2.1. Sample Preparation

**Figure 1:**
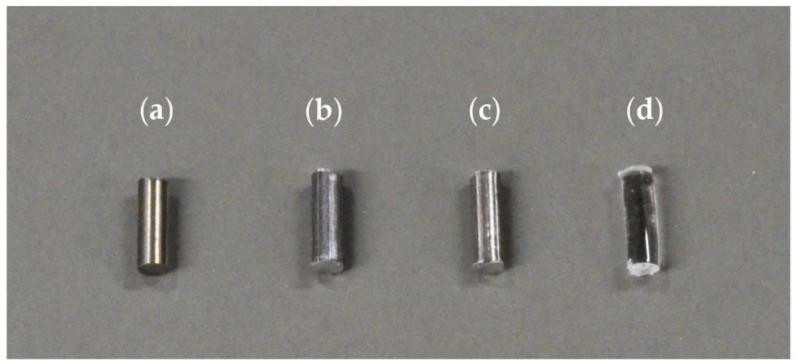
Photograph of prepared implant samples. (**a**) titanium alloy (TiA): American Society for Testing and Materials (ASTM) B348 Gr5 (Ti–6Al–4V) titanium alloy, (**b**) magnesium alloy (MgA) AZ31 magnesium alloy, (**c**) Mg: pure magnesium, (**d**) poly-l-lactic acid (PLA): ISO 13,485 compliant medical poly-l-lactic acid. Each material was processed into a cylindrical shape with a diameter of 1.6 mm and a length of 4.0 mm.

American Society for Testing and Materials (ASTM) B348 Gr5 (Ti–6Al–4V) titanium alloy (TiA) (Nilaco Corporation, Tokyo, Japan), AZ31 magnesium alloy (MgA) (Osaka Fuji Corporation, Amagasaki, Japan), pure Mg (Osaka Fuji Corporation, Amagasaki, Japan), and medical-grade (according to ISO 13485) poly-L-lactic acid (PLA) (BMG Inc., Kyoto, Japan) were used (Figure 1). Unthreaded and uncoated smooth wires of diameter 1.6 mm were purchased from each manufacturing company. The wires were processed into cylinders 4.0 mm in length, using a low-speed, water-cooled diamond saw (Isomet 11-1280; Buehler, Lake Bluff, IL, USA). Burrs were removed from the cross-section, and the surface was smoothened via polishing. The cylinders were lightly cleaned ultrasonically with distilled water and sterilized with ethylene oxide gas. The PLA filament was delivered with an accuracy of 1.6 mm ± 20% for manufacturing technology purposes. Therefore, after processing to a length of 4.0 mm, samples that deviated from a diameter of 1.6 ± 0.2 mm were excluded. Table 1 shows the compositions of TiA, MgA and Mg. TiA was 6.22% aluminum, 4.13% vanadium and Ti. MgA was 97.96% Mg, 1.08% Al and 0.62% Zn. The Mg was 99.97% pure. The fluorescent X-ray analyzer (JSK-3220ZS; JEOL Ltd., Tokyo, Japan) cannot identify elements lighter than sodium, and so was it unsuitable for the analysis of PLA.

### 2.2. Nanoindentation Testing of the Mechanical Properties of the Implants

The four implant materials were encapsulated in epoxy resin (Epofix; Struers, Copenhagen, Denmark) for nanoindentation testing and cut in the longitudinal direction of the implant. Then, the cross sections were polished using diamond suspensions (0.25-, 1-, and 3-μm particle sizes) to obtain a surface suitable for nanoindentation. The specimens were washed with distilled water and lightly cleaned ultrasonically. Nanoindentation testing of the surface of the specimens (ENT-1100a; Elionix Inc., Tokyo, Japan) was carried out at 28 ± 0.1 °C with a peak load of 100 mN using a Berkovich indenter (n = 10). Hardness and elastic modulus values were calculated using the ENT-1100a software.

### 2.3. In Vitro Immersion Test

The implant materials were immersed in simulated body fluid (SBF) with a temperature of 37 °C (Table 2 [34]) for up to 14 days. The pH of the SBF was adjusted to 7.4 using 1 M HCl. The pH of the medium was monitored daily using a pH meter (F-72; Horiba Ltd., Kyoto, Japan). The materials were allowed to dry in air, and changes in their weights were noted. The degradation rate (*DR*; mm/year) was calculated based on the following equation according to ASTM-G31-72 [35]:

*DR* = 3.65*W*/*ATD* (3)

where *T* is the immersion time (days), *W* is the weight loss after *T* hours of immersion (mg), *A* is the initial surface area (cm^2^), and *D* is the density of the material (Ti, 4.43 g/cm^3^; MgA, 1.78 g/cm^3^; Mg, 1.74 g/cm^3^; and PLA, 0.98 g/cm^3^).

### 2.4. Animal Experiments

#### 2.4.1. Animal Model and Experimental Design

The animal experiments were approved by the Animal Experiment Board of the Health Sciences University of Hokkaido (approval number, 87). Fifty-one male Wistar rats aged 7 weeks (body weight, 149.0–181.7 g) were used. Rats that did not exhibit any abnormality after quarantine breeding for one week at the Animal Experiment Center of the Health Sciences University of Hokkaido were used. The quarantine breeding and breeding during the experimental period were carried out with ad libitum access to water and rat chow (certified diet MF for experimental animals; Oriental Yeast Co., Ltd., Tokyo, Japan) in a breeding environment with a 12-h daily light cycle. One TiA, MgA, Mg, or PLA sample was implanted into each femoral bone. The experimental periods were 2, 4 and 12 weeks, and 17 rats were randomly assigned to each period. The rats were subjected to clinical observation daily and their body weight was measured at regular intervals.

Two rats died intraoperatively due to deep anesthesia. When removing the femur after euthanasia, a fracture was found around one PLA implant. Detachment of the implant from the bone occurred in one rat in each of the TiA and Mg groups. Finally, 54 femoral samples were used for micro-CT and biomechanical push-out testing, 18 for scanning electron microscopy (SEM) and 23 for histological evaluation.

#### 2.4.2. Surgical Procedure

**Figure 2:**
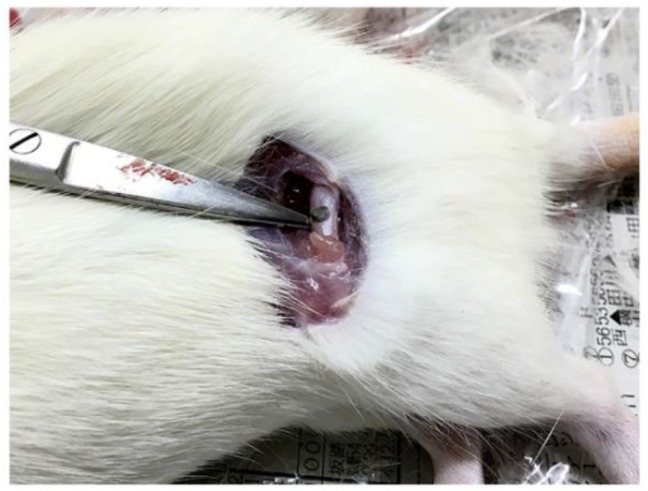
Photograph during the surgical procedure. The exposed femur and the inserted implant are shown at the tip of the instrument.

The rats were anesthetized first via inhalation of isoflurane (isoflurane inhalation anesthetic solution; Pfizer Japan Inc., Tokyo, Japan), then 0.125 mL/100 g of a mixture of medetomidine hydrochloride (10 mL Domitol^®^; Nippon Zenyaku Kogyo Co., Ltd., Koriyama, Japan), midazolam (10 mg; Teva Takeda Pharma Ltd., Nagoya, Japan), and butorphanol tartrate (5 mg Vetorfar^®^; Meiji Seika Pharma Co., Ltd., Tokyo, Japan) were injected intraperitoneally. The mixture was prepared by mixing 3.0 mL of medetomidine hydrochloride, 8.0 mL of midazolam, 10.0 mL of butorphanol tartrate and 4.0 mL of physiological saline. The skin, fascia and periosteum were exfoliated sequentially, and the mid-diaphyseal region of the femur was exposed (Figure 2). A dental round-bar and a non-taper fissure-bar were used to prepare the implantation bed vertically to the long axis of the femur. Drilling was performed at low rotational speed, and profuse physiological saline irrigation was applied via a syringe to minimize frictional heat and thermal necrosis. The cylindrical implant was inserted by gentle tapping, resulting in a uniform press fit. The operating field was irrigated thoroughly with physiological saline, and the wound was closed in layers using a suture needle (surgical weak square needle 3/8 circle, spring hole No. 0; Natsume Seisakusho Co., Ltd., Tokyo, Japan) and a suture (nylon suture 5-0; Natsume Seisakusho Co., Ltd.). Thereafter, the contralateral side was operated on in the same way using the same implant type. The general anesthesia was antagonized via intraperitoneal injection of atipamezole hydrochloride (Antisedan^®^; Nippon Zenyaku Kogyo Co., Ltd., Koriyama, Japan). Postoperatively, 0.5 mL of sulpyrine hydrate (sulpyrine injection 250 mg NP; Nipro Pharma Corporation, Osaka, Japan) was added per 400 mL of drinking water for two weeks as an anti-inflammatory and analgesic.

#### 2.4.3. Sample Extraction

Volatile isoflurane (Pfizer Japan Inc.) was administered to the rats to induce general anesthesia. The rats were next euthanized using CO_2_ gas. Thereafter, the heart was punctured, and blood was aspirated for the evaluation of systemic inflammation using saline containing 5.0 mL of anticoagulant (heparin sodium injection; Shimizu, Ajinomoto Pharmaceuticals Co., Ltd., Tokyo, Japan), and perfusion fixation was performed using a tissue fixative (10% neutral-buffered formalin solution; Kanto Chemical Co., Inc., Tokyo, Japan). Bilateral femurs containing implant samples were excised from each rat. Immediately after harvest, all soft tissues were carefully removed.

The bone implant specimens were immersed in the tissue fixative (10% neutral-buffered formalin solution; Kanto Chemical Co., Inc., Tokyo, Japan) in airtight polyethylene tubes and stored at 4 °C.

### 2.5. Three-Dimensional High-Resolution Micro-CT

**Figure 3:**
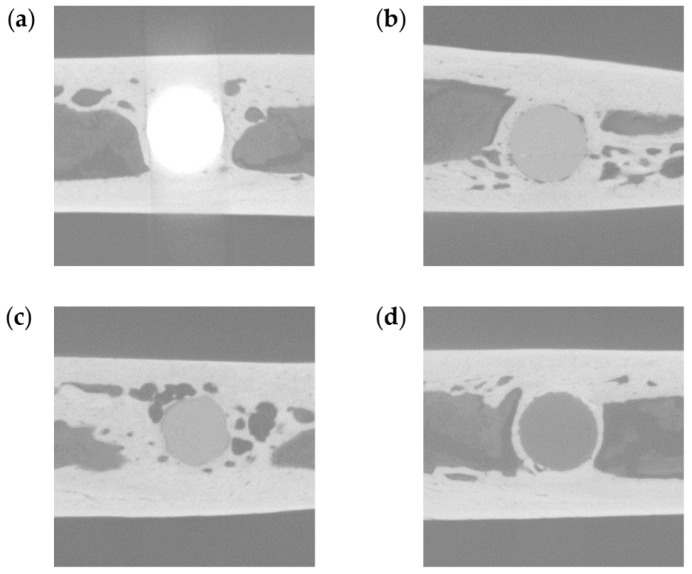
Representative micro-computed tomography (CT) images. (**a**) TiA, (**b**) MgA, (**c**) Mg, and (**d**) PLA implants.

The bone implant samples were scanned at room temperature using an inspeXio SMX-225CT system (Shimadzu Corporation, Kyoto, Japan) at a voxel size of 7 μm. To scan the TiA, MgA, Mg and PLA samples (which have different X-ray absorption coefficients) and cortical and cancellous bones without excess and deficiency, the system was operated at 70 kV, with a current of 160 μA and an integration time of 125 ms (Figure 3). A brass plate of 0.1-mm thickness was attached in front of the X-ray source to reduce beam hardening noise, which hampers analysis. The evaluation was conducted with the pins parallel to the rotation axis of the microCT, resulting in approximately 900–950 slices per sample. For each sample, 14 TiA, 12 MgA, 12 Mg and 16 PLA samples were used for micro-CT analysis. Of these, 18 were in two weeks, 17 in four weeks and 19 in 12 weeks.

The micro-CT data were analyzed using TRI/3D-BON software (Ratoc System Engineering Co., Ltd., Tokyo, Japan). The hydroxyapatite reference phantom (U5D 1 mmH; Ratoc System Engineering Co., Ltd.) was scanned under the same conditions as the samples, and a calibration curve was created from the obtained CT values to calibrate the data. The implant images were automatically segmented at different thresholds due to their different densities and the absorption of X-rays (Table 3). The extraction level gives the center of the thresholds, the extraction width gives the width from the extraction level—i.e., they give the upper and lower thresholds for the segmentation. Bone tissues that were formed via the combination of cortical and trabecular bone were automatically extracted. The noise generated by automatic extraction (i.e., areas extracted unexpectedly) was removed using a Boolean operation. Specifically, the command was executed in the order of one voxel for Erosion, one voxel for Dilation, two voxels for Dilation, and two voxels for Erosion.

The bone density (BV/TV, %) and bone-implant contact (BIC, %) in the area of the peri-implant bone were determined [36]. BV/TV is the ratio of bone volume (BV) to total volume (TV). A circle with a diameter 0.5 mm greater than that of the implants was selected as the region of interest (ROI) for the evaluation of BV/TV. BIC represents the surface area of bone tissue in the ROI. The ROI for the evaluation of BIC was defined as a one-voxel-thick ring that was two voxels from the implant surface.

### 2.6. Biomechanical Push-Out-Testing

**Figure 4:**
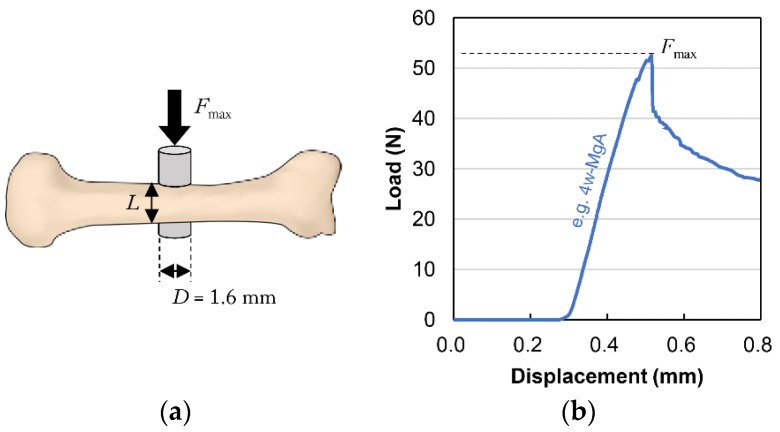
Schematic diagram of biomechanical push-out testing. (**a**) *F*_max_ is the maximum push-out force (N), *L* is the mean length of bone in contact with the implant (mm), and *D* is the diameter of the implant (1.6 mm). The ultimate shear strength of the interface (*τ*_u_, N/mm^2^) was calculated by dividing the maximum push-out force by the total bone-implant contact area (*BIC*_Cg_ = *πDL*). (**b**) Typical load-displacement curve (e.g., 4-week sample from the MgA group).

The extracted femur samples were returned to room temperature and subjected to biomechanical push-out testing using a table-top universal testing instrument (EZ Test; Shimadzu Corp., Kyoto, Japan). A tensile test jig (Shimadzu Corp., Kyoto, Japan) was applied to the location where each implant sample was fixed to the femur to enable accurate alignment with the strictly perpendicular axial push-out. Each specimen was loaded at a constant displacement rate of 1.0 mm/min using a load cell (range of force, 0–500 N; precision, ±1.0%) until failure occurred. The displacement was constantly recorded using Trapezium 2 software (Version 2.35, Shimadzu Corp., Kyoto, Japan). Throughout the test, the samples were kept moist via the frequent application of physiological saline. The maximum push-out force (*F*_max_) was determined from the load-displacement curves (Figure 4b). The ultimate shear strength of the interface (*τ*_u_, N/mm^2^) was calculated by dividing the maximum push-out force by the total BIC area (*BIC*_Cg_) using the following two equations:

*τ*_u_ = *F*_max_/*BIC*_Cg_ (4)

*BIC*_Cg_ = *πDL* (5)

where *D* is the diameter of the implant sample (1.6 mm) and *L* is the mean length of bone in contact with the implant (mm) (Figure 4a). *L* was measured five times for each specimen using Vernier calipers (standard caliper M type N-15; Mitutoyo Corporation, Kawasaki, Japan; measurement range, 0–150 mm; accuracy, 0.05 mm) before biomechanical push-out testing.

### 2.7. Scanning Electron Microscopy

After biomechanical push-out testing, the surface morphology of the bone implant samples was examined using scanning electron microscopy (SEM; JMS-6610LA; JEOL Ltd., Tokyo, Japan). The samples were sputter-coated with pure gold (SC-701 AT; Sanyu Electron Co., Ltd., Tokyo, Japan) and examined at 15 kV. Additionally, elemental mapping analysis using energy dispersive X-ray spectroscopy (EDX) was performed to characterize the degradation layer on the implant samples. The working distance was 10 mm.

The bone implant samples were encapsulated in epoxy resin (Epofix). After 24 h, the specimens were cut perpendicularly to the axial direction at the site encompassing the implant using a low-speed, water-cooled diamond saw (Isomet; Buehler). The specimens were lightly ground with 600-grit sandpaper, polished using diamond suspensions with particle sizes of 3 and 1 μm, and polished again using aluminum oxide suspensions with a particle size of 0.25 μm (Buehler). The resulting specimens were examined using SEM and EDX to characterize the bone-implant interface. For each sample, 3 TiA, 6 MgA, 5 Mg and 4 PLA samples were used for SEM and EDX to characterize the bone-implant interface. Of these, five were in 2 weeks, six in 4 weeks and seven in 12 weeks.

### 2.8. Histological Evaluation

#### 2.8.1. Preparation of Non-Decalcified Polished Specimens

The retrieved femora were embedded and polymerized in methyl-methacrylate resin (Regolac^®^; Okenshoji Co., Ltd., Tokyo, Japan) in accordance with the manufacturer’s instructions. Subsequently, to obtain cross-sections of the implant and the surrounding bone tissue, the samples were cut using a diamond band saw (BS3000; Exakt, Hamburg, Germany) into 50-μm slices perpendicular to the long axis of the implant. The specimens were next lightly ground with 600-grit sandpaper, polished using diamond suspensions with particle sizes of 3 and 1 μm, and polished again using an aluminum oxide suspension with a particle size of 0.25 μm (Buehler).

#### 2.8.2. Observation of Toluidine Blue-Stained Sections

The sections were stained with toluidine blue according to a standard protocol and visualized using light microscopy (S2H10·BH2; Olympus Corporation, Tokyo, Japan), with a focus on new bone formation and the contact between the implant surface and the surrounding bone, excluding intervening connective tissue. For each sample, 6 TiA, 6 MgA, 6 Mg and 5 PLA samples were used for micro-CT analysis. Of these, seven were in 2 weeks, eight in 4 weeks and eight in 12 weeks.

### 2.9. Blood Testing

To evaluate systemic inflammation, 2 mL of blood was collected in a standard serum vial, incubated at 25 °C for 30 min, and left at 4 °C for 16 h to coagulate. The clot was centrifuged at 1200× *g* for 30 min. The supernatant was removed and stored at −20 °C. A Rat IL-6 enzyme-linked immunosorbent assay (ELISA) kit (Invitrogen BMS625; Thermo Fisher Scientific Inc., Waltham, MA, USA) was used to determine the serum levels of interleukin-6 (IL-6) according to the manufacturer’s instructions. The absorbance of each well was measured using an absorbance meter (Infinite^®^ F200; Tecan Trading AG, Männedorf, Switzerland; primary wavelength, 450 nm; reference wavelength, 620 nm). A standard curve was created from the mean absorbance of each standard concentration, which was used to convert absorbance values to concentrations (pg/mL) (Excel 2016; Microsoft, Redmond, WA, USA).

### 2.10. Statistical Analysis

Statistical analysis was performed using SPSS 25.0 software (IBM, Armonk, NY, USA). Mean values were compared using one-way analysis of variance (ANOVA) followed by the Tukey test. The statistical analysis was performed between different time points of the same materials and different materials at the same time points. The *p*-values < 0.05 were taken to indicate statistical significance.

## 3. Results

### 3.1. Mechanical Properties

**Figure 5:**
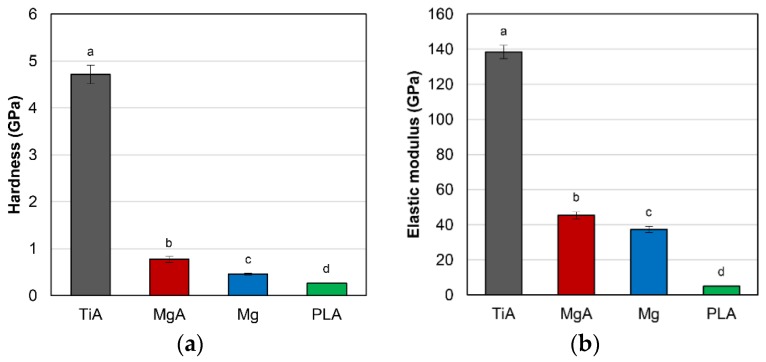
(**a**) Hardness (GPa) and (**b**) elastic modulus (GPa) of the implants as determined by nanoindentation testing. Identical letters indicate no significant difference (*p* < 0.05, Tukey’s test).

Figure 5 shows the results of nanoindentation testing. The mean hardness was 4.72 GPa for TiA, 0.77 GPa for MgA, 0.46 GPa for Mg and 0.26 GPa for PLA (Figure 5a). The mean elastic modulus was 138.45 GPa for TiA, 45.41 GPa for MgA, 37.35 GPa for Mg and 4.89 GPa for PLA (Figure 5b). MgA exhibited a significantly lower hardness and elastic modulus than TiA, but significantly greater mechanical properties than Mg or PLA. The mean hardness and elastic modulus values of MgA were, respectively, approximately 1.7- and 1.2-fold those of Mg.

### 3.2. In Vitro Immersion Testing

**Figure 6:**
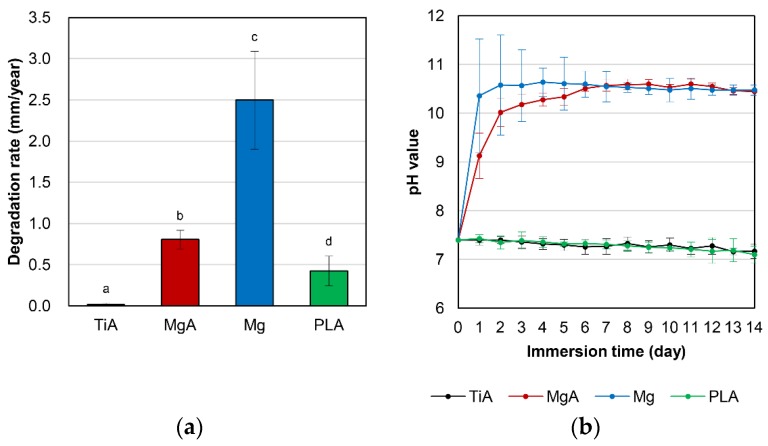
(**a**) Degradation rate (mm/year) and (**b**) pH change in the solution when samples were immersed in simulated body fluid (SBF) for 14 days. Identical letters indicate no significant difference (*p* < 0.05, Tukey’s test).

Figure 6a shows the mean degradation rate as determined by the immersion test. The mean degradation rate was 0.02 mm/year for TiA, 0.81 mm/year for MgA, 2.50 mm/year for Mg and 0.43 mm/year for PLA. The mean degradation rate of Mg was significantly greater than those of the other three materials. The degradation rate of MgA was approximately twofold that of PLA.

The pH variation in the SBF during immersion testing is shown in Figure 6b. MgA and Mg produced an extremely high pH from day 1. By day 6, Mg produced a higher pH than MgA; thereafter MgA and Mg stabilized at pH 10.5–10.6.

### 3.3. Animal Tests

No abnormal changes such as redness, swelling, or suppuration were observed in any of the rats. The mean body weight at the time of euthanasia did not differ significantly among the groups or experimental periods (data not shown).

### 3.4. Three-Dimensional High-Resolution Micro-CT

**Figure 7:**
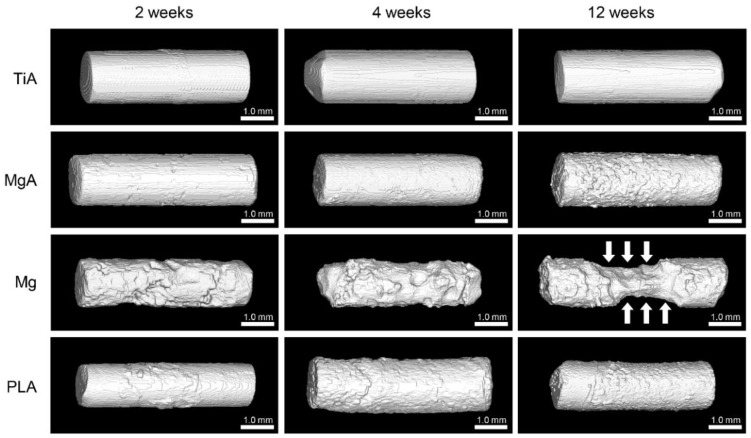
Three-dimensional reconstructions of implants based on the micro-computed tomography (CT) data. No changes were observed in the TiA group. Absorption from the surface layer, which increased over time, was observed in the MgA, Mg and PLA groups. Marked absorption at the portion in contact with the bone marrow (white arrow) at 12 weeks was observed in the Mg group.

Figure 7 shows the three-dimensional reconstructions of the implants based on the micro-CT data. No change in surface morphology was observed in the TiA group; however, MgA, Mg and PLA were absorbed in a time-dependent manner. In particular, the area of Mg in contact with bone marrow (Figure 7, white arrows) was extremely thin at 12 weeks.

**Figure 8:**
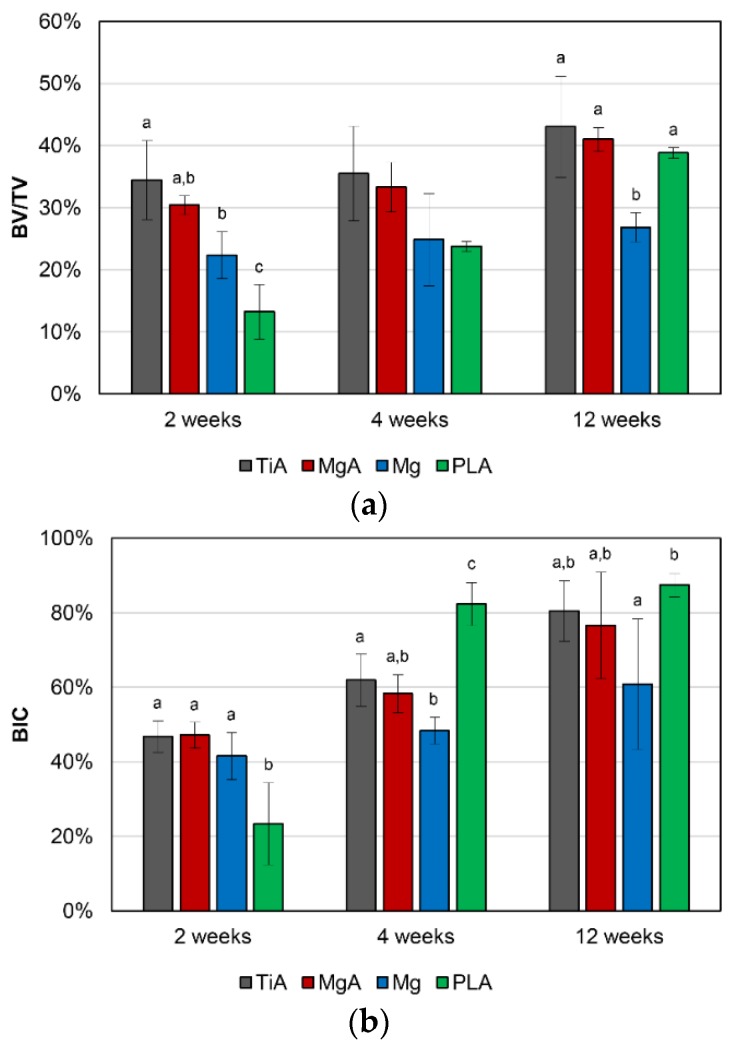
(**a**) Bone density (BV/TV, %) and (**b**) bone-implant contact (BIC, %) as determined by micro-CT. No significant differences were observed between the TiA and MgA groups. Identical letters indicate no significant difference (*p* < 0.05, Tukey’s test).

Figure 8 shows the mean BV/TV and BIC values obtained via micro-CT. The mean BV/TV values increased significantly over time (Figure 8a). There were no significant differences between TiA and MgA in all experimental periods. There was also no significant difference in the mean BV/TV values between the MgA and Mg groups at 2 weeks; however, the mean BV/TV value of the MgA group was significantly higher than that of the Mg group at 4 and 12 weeks. The mean BV/TV value of the PLA group was significantly lower than that of the MgA group at 2 weeks; however, there was no significant difference in the mean BV/TV values between the MgA and PLA groups at 4 and 12 weeks.

The mean BIC values increased over time (Figure 8b). There was no significant difference in the mean BIC values between the TiA and MgA groups at all experimental periods. There was no significant difference in the mean BIC values between the MgA and Mg groups at all experimental periods. Initially, lower BIC values were observed in the PLA group compared to the other groups, but higher BIC values were observed at 4 weeks. Finally, there were no significant differences in the BIC values among the TiA, MgA and PLA groups (12 weeks).

### 3.5. Biomechanical Push-Out Testing

**Figure 9:**
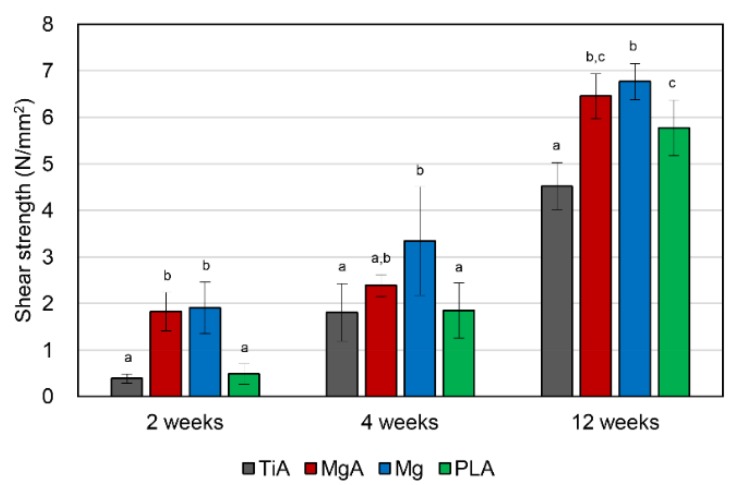
Shear strength of the bone-implant interface as determined using biomechanical push-out testing (N/mm^2^). Significantly higher shear strength was observed in the MgA group compared to the TiA group. Identical letters indicate no significant difference (*p* < 0.05, Tukey’s test).

The shear strength of the interface is shown in Figure 9. The shear strength of the interface increased significantly over time in all groups. The mean shear strength of the MgA group was significantly higher than that of the TiA group at 2 and 12 weeks. The shear strength of the Mg group at 2 and 12 weeks was similar to that of the MgA group. Similar shear strengths were observed in the PLA and TiA groups at 2 weeks. The shear strengths observed in both groups increased to a level similar to that observed in the MgA group at 12 weeks. However, the values in the latter group increased thereafter, and became significantly higher than those values observed in the TiA group.

### 3.6. SEM

#### 3.6.1. Implant Surface

**Figure 10:**
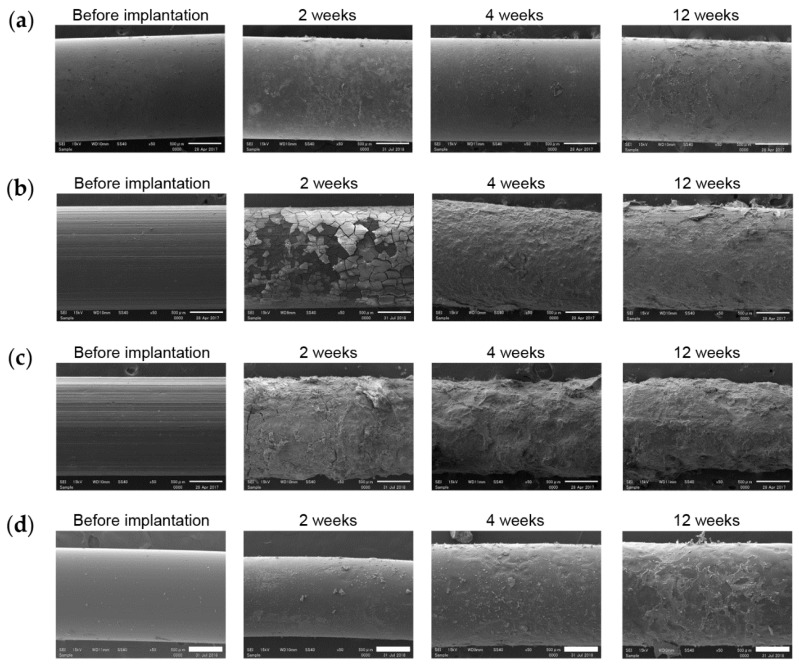
Representative scanning electron microscopy (SEM) images of the implant surface removed during biomechanical push-out testing. (**a**) TiA, (**b**) MgA, (**c**) Mg and (**d**) PLA implants. SEM image, 100× magnification. Scale bars at lower right, 500 μm.

Figure 10 shows SEM images of the implant surfaces observed during biomechanical push-out testing. In the TiA group, Ti-containing substances were observed to cover the implant surface (Figure 10a). However, a set of calcium and phosphorus, the main components of bone, were not found at the corresponding positions. The horizontal lines on the surface of the MgA implant before implantation were scratches generated during manufacturing (Figure 10b). At 2 weeks, many cracks were observed on the surface, and the above-mentioned scratches were present. Calcium and phosphorus were detected on the surface by EDX, but little Mg was observed (data not shown). At 4 and 12 weeks, the surface of the crack had peeled off. At the peeled-off site, internal Mg, but not calcium and phosphorus, was detected (data not shown). In addition, oxygen was detected at the position with internal Mg. Similar changes were observed in the Mg group (Figure 10c). However, the surface layer was peeled off and the scratches were absent at 2 weeks, suggesting that Mg degraded more rapidly than MgA. The diameter of the implant decreased over time. There were no cracks on the surface of the PLA implant, which was smoothly degraded (Figure 10d). Although the calcium and phosphorus levels were lower than in the MgA and Mg groups, some bone-like debris was identified at 12 weeks. The PLA implants did not have a uniform diameter, because the PLA filament had a diameter of 1.6 mm ± 20%.

#### 3.6.2. Bone-Implant Interface

**Figure 11:**
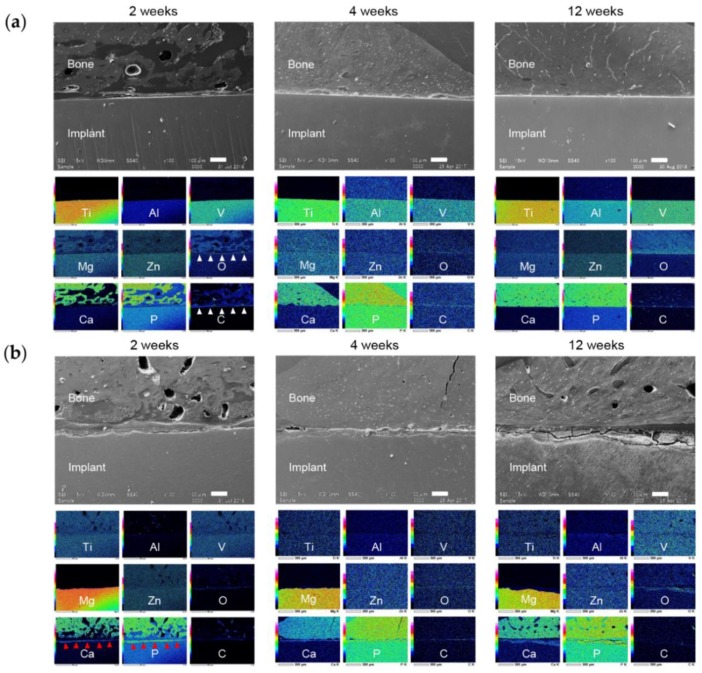
Representative SEM images and energy dispersive X-ray spectroscopy (EDX) elemental mapping of the bone-implant interface. (**a**) TiA and (**b**) MgA implants. SEM image, 100× magnification. Scale bars at lower right, 100 μm. EDX elemental mapping corresponds to the SEM image. White arrowheads indicate fibrous tissue (existence of O and C), red arrowheads indicate new bone formation (existence of Ca and P).

**Figure 12:**
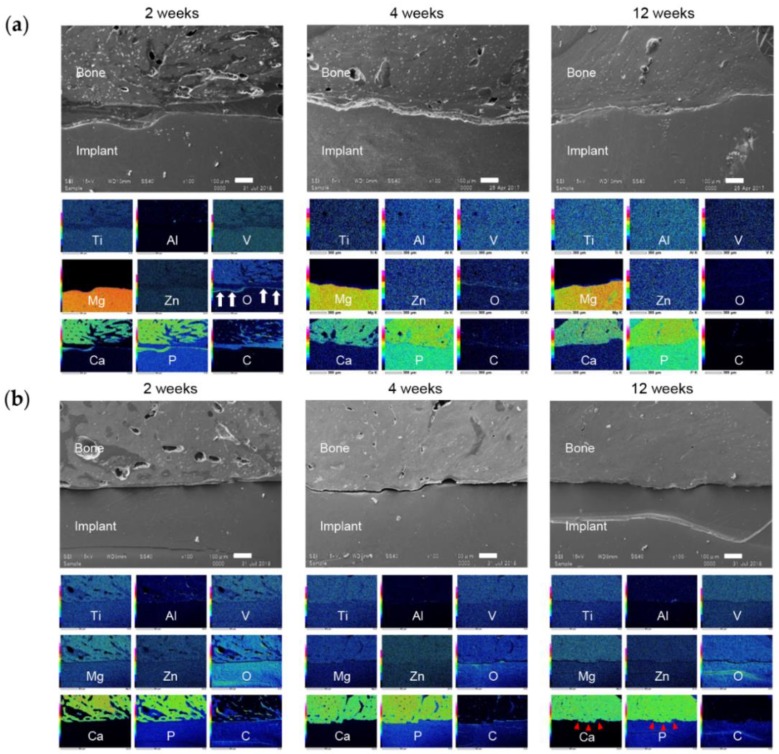
Representative SEM images and energy dispersive X-ray spectroscopy (EDX) elemental mapping of the bone-implant interface. (**a**) Mg and (**b**) PLA implants. SEM image, 100× magnification. Scale bars at lower right, 100 μm. EDX elemental mapping corresponds to the SEM image. White arrows indicate the oxide layer of the Mg implant, red arrowheads indicate new bone formation (existence of Ca and P).

SEM images of the bone-implant interface are shown in Figure 11. In the TiA group there was a gap at the interface at 2 weeks, and a layer containing carbon and oxygen (Figure 11a). At 4 and 12 weeks, the gap had narrowed. Degradation of the implant surface is visible at 2 weeks in the MgA group (Figure 11b). A layer containing calcium and phosphorus was observed on the surface of the degraded implant; this layer had increased in thickness at 4 and 12 weeks. The Mg group exhibited greater degradation than the MgA group, as evidenced by large surface irregularities after 2 weeks (Figure 12a). Oxygen was detected in the area corresponding to the implant surface (Figure 12a, white arrows). At 2 weeks, a calcium- and phosphorus-rich layer had formed upon the surface of the degraded implant. In the PLA group, surface degradation proceeded relatively smoothly (Figure 12b). A calcium and phosphorus layer with the same strength as bone tissue was detected at the degraded site (Figure 12b, red arrowhead).

### 3.7. Histological Evaluation

**Figure 13:**
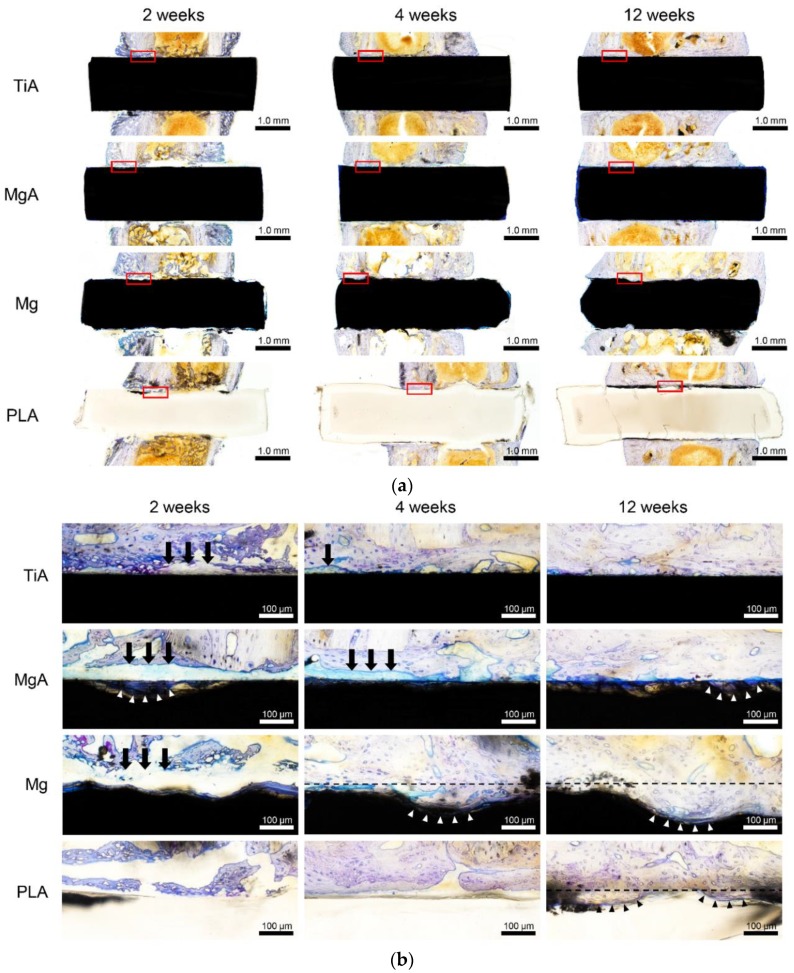
Representative toluidine blue-stained image of bone tissue surrounding the implants. (**a**) Low and (**b**) high magnification images; the red box in (**a**) is magnified in (**b**). In the markedly degraded sample, the black broken line indicates the initial interface. Arrows indicate fibrous tissue between implant and bone, arrowheads indicate new bone formation to the absorbed implant area.

Figure 13 shows toluidine blue-stained histological images of the implants. The red box in the weakly magnified image (Figure 13a) corresponds to the strongly magnified image (Figure 13b). Contact of implant and bone was visually confirmed for all samples at 12 weeks. In several samples, fibrotic tissue was observed at the bone-implant interface (Figure 13b, black arrows). However, the amount of fibrotic tissue decreased over time, resulting in an increased BIC area. In the MgA group, the stained area at the site of bone resorption suggested new bone formation (Figure 13b, white arrowhead). This finding is consistent with those of the SEM analysis. The highest degradation rate was observed in the Mg group, and new bone formation did not delay the degradation (Figure 13b, white arrowhead). Morphology associated with relatively smooth degradation was observed in the PLA group, and new bone formation was observed at the area of bone resorption (Figure 13b, black arrowhead).

### 3.8. Blood Parameters

**Figure 14:**
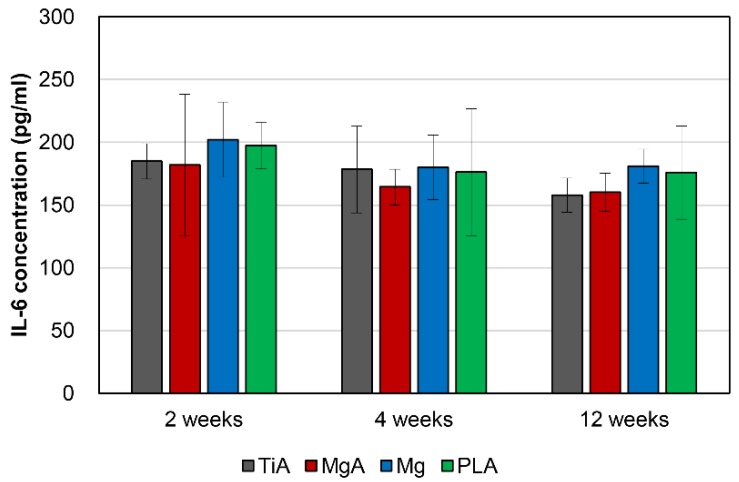
Serum interleukin (IL)-6 concentrations (pg/mL). The IL-6 levels were normal and similar across all samples, indicating the absence of a systemic inflammatory response. One-way analysis of variance (ANOVA) and Tukey’s test were conducted to compare sample values (*p* < 0.05).

Serum IL-6 levels were normal, indicating the absence of a systemic inflammatory response, and was statistically similar among groups (Figure 14).

## 4. Discussion

### 4.1. Characteristics of the Implant Materials

MgA implants had a hardness approximately fivefold lower, and an elastic modulus approximately threefold lower, than TiA implants. By contrast, MgA implants had a hardness threefold higher and an elastic modulus ninefold higher than PLA implants. Mg has significantly greater mechanical properties than PLA. The AZ31 MgA contained 1.08% Al and 0.62% Zn. MgA implants had a hardness approximately 1.7-fold and an elastic modulus approximately 1.2-fold that of Mg implants. This is consistent with a report that the addition of Al, Si, Sn, Zn and Zr improves the strength of Mg [20].

Pure Mg corrodes rapidly in vivo [34,35], which must be controlled for use in implants [16]. Mg samples decomposed at a significantly higher rate than MgA samples. Thus, alloying Mg with Al and Zn improved the corrosion resistance to SBF. Similarly, Gu et al. reported that the addition of Al, In, Mn, Zn, or Zr to Mg reduced the corrosion rate in SBF and Hank’s solution [20].

The pH of the solution in the MgA and Mg groups increased significantly after day 1 of the immersion test. The corrosion of metals involves the anodic reaction, in which the metal is ionized, and the cathodic reaction, in which H^+^ and oxygen are reduced while maintaining electrical neutrality [15]. Mg-based materials immersed in SBF are corroded and dissolved by water based on the Equations (1) and (2). The increase in solution pH was likely due to the generation of hydrogen gas and hydroxide ions (OH^−^). The solution with Mg had a higher pH than the solution with MgA until day 6, likely due to greater OH^−^ generation caused by the higher degradation rate of the former.

The greater corrosion of Mg samples compared to MgA samples was confirmed via in vitro immersion testing and micro-CT analysis. In the EDX of the bone implant interface in vivo, the oxygen-rich layer in the area corresponding to the surface Mg layer was thicker in the Mg group than in the MgA group. This was probably because the greater corrosion of Mg promotes the formation of a thicker and deeper oxide layer.

The degradation layer was not intentionally removed. The issue with measuring the weight loss without removing the degradation layer lies in the fact that the precipitation of degradation products on the different materials may be different. It also impairs the comparison to other in vitro studies. Unfortunately, the degradation layer was included Mg, and plays an important role in the functioning of the implant for clinical use; e.g., the effect of the implant being fixed to the bone through the degradation layer. Therefore, weight loss was calculated using the weight including the degradation layer intentionally.

The content of Fe in the pure Mg sample may lead to the formation of galvanic cells, thus leading to high degradation rates. Normally, pure Mg does not contain Fe, so it may be a cutting piece generated during manufacturing. In any case, the effect on corrosion is considered to be negligible because it is very small, 0.03 wt %.

The reason that PLA was selected as a comparison of absorbable metal materials in this research was that it is currently the main absorbable material used in clinical practice. However, the findings were self-evident that PLA had lower mechanical properties than absorbable metal materials, and its degradation characteristics were not due to corrosion.

### 4.2. Osseointegration

There was no significant difference in BV/TV and BIC values between the TiA and MgA groups at all experimental periods. By contrast, the BV/TV and BIC values of the MgA group were significantly higher than those of the PLA group at 2 weeks after implantation. Therefore, the bone response of MgA is superior to that of PLA. Witte et al. reported that Mg alloys result in a significantly greater area of calcified bone around implants and a higher mineral deposition rate than PLA [17]. The same authors evaluated remodeling around AZ91D MgA implants and autologous bone implants at 3–6 months after implantation in rabbits [29]. Histomorphometry showed that the bone mass was greater, and the bone tissue more mature, around the MgA than the autologous bone implants. These studies did not quantitatively assess the bone response of MgA. However, Mg enrichment reportedly promotes bone formation [37], enhances osteoblast adhesion [38,39,40], temporarily inhibits osteoclast activity, and regulates signal transduction pathways associated with human bone-derived cells [19].

The MgA implant surface had begun to degrade at 2 weeks, and the surface harbored a layer containing calcium and phosphorus. Histologically, a stained area resembling existing bone was found at the site of resorption, suggesting new bone formation. This indicates that new bone began to form from the implant surface, not the existing bone, at 2 weeks. Similar observations as those of the MgA group were made for the Mg group. New bone formation occurred without delay in the Mg group, which showed relatively fast degradation.

Janning et al. reported that the local increase in Mg ion concentration that accompanies Mg(OH)_2_ dissolution causes localized alkalosis [19]. Although micro-CT and histological results showed that Mg exhibits osteoinductive activity, the underlying mechanism is unknown.

It has been suggested that the non-uniform diameters of PLA implants may affect BIC and BV/TV measurements if the fit was worse during implantation. However, the results of BV/TV and BIC showed that PLA increased significantly with an increasing implantation period. Thus, the non-uniform diameter of the PLA implant did not have a significant effect in this study.

### 4.3. Surface and Absorption Characteristics

As described above, Mg causes corrosion and deterioration in the body based on the Equations (1) and (2). The pH was increased by hydroxide ions (OH^−^) generated by the corrosion and dissolution of the Mg surface, leading to local alkalosis. The increased pH decreased the solubility of the corrosion product Mg(OH)_2_, which precipitated on the implant surface. Corrosion can be inhibited by covering the implant surface with Mg(OH)_2_. The precipitation of Mg(OH)_2_ is also influenced by the amount of Mg^2+^ near the surface of the Mg implant. Therefore, the Mg corrosion rate is increased by removing Mg^2+^ and OH^−^ from the implant surface. That is, in an environment with marked blood flow, such as in blood vessels and bone marrow, the corrosion rate is higher than in areas with slower blood flow, such as in cortical bone and subcutaneous tissue. In addition, if new bone is formed on the surface of the Mg implant, the coating will reduce the area exposed to fluid reflux, further reducing the corrosion rate. The three-dimensional reconstruction images based on the micro-CT data showed that the part in contact with the bone marrow was thin. By contrast, corrosion and degradation were slow in areas in contact with cortical bone, likely due to fluid circulation.

The correlation between in vitro and in vivo corrosion rates is unclear [41,42,43]. Although there was a correlation between the in vivo and in vitro degradation rates, the corrosion rate of the Mg implant differed markedly, depending on the site, due to the influence of the in vivo environment. Johnston et al. reported that large variations in in vitro corrosion rates hamper in vitro and in vivo comparisons [43]. Therefore, reproducible and standardized methods are needed to enable more accurate assessment of corrosion rates in vitro [16].

SEM and EDX analyses revealed that calcium and phosphorus were enriched on the surface at 2 weeks after implantation. Mg was not detected after 2 weeks, suggesting the formation of a relatively thick calcium phosphate layer. At 4 weeks, the surface was exfoliated during mechanical push-out testing, leading to the loss of calcium and phosphorus, and internal Mg was detected. In addition, oxygen was detected at the corresponding position, indicating the presence of Mg(OH)_2_.

More calcium and phosphorus were detected at the surface layer of MgA and Mg implants than the surface layer of the TiA implants. Due to the corrosion of Mg, Mg-based materials are superior to Ti alloys, not only in terms of the bone response, but also surface roughness. By contrast, calcium and phosphorus were not detected in the PLA group, in which implants exhibited a smooth surface, suggesting that there was less degradation of surface roughness than in the MgA and Mg groups. Therefore, surface roughness must be increased to enhance mechanical bonding.

### 4.4. Inflammatory Response and Biocompatibility

There was no dissolution of the peri-implant bone, which can lead to implant loosening or local inflammation. No clinical signs of local inflammation (redness, swelling, drainage, etc.) were observed. There were no significant differences in serum IL-6 concentrations among rats treated with four types of implants, and serum concentrations were normal in all of the rats.

In this experimental design, a large number of femoral samples with implants were used for various experiments. The femurs on both sides were used to save any cost and animal life. Strictly speaking, the sample for the evaluation of systemic inflammation suggests that surgery with only one femur was more accurate.

As Mg corrodes and degrades, the eluted Mg^2+^ is excreted in the urine. The plasma Mg concentration is maintained until the renal filtration capacity is exceeded [11]. Zhang et al. investigated the decomposition of Mg–Zn–Mn alloys in vivo, and reported that it does not cause damage to the liver and kidneys, nor significantly alter the serum concentrations of inorganic ions [44]. Other studies of Mg–Ca [45] and Mg–Zn [46] alloys have reported similar results. Since some EDX images suggest that small amounts of Mg are present in the bone, small amounts of Mg may be deposited in the bone matrix. Further studies are needed to determine whether Mg has the property of depositing in bone during metabolism.

Upon histological analysis, no inflammatory cell infiltration and only temporary fibrous tissue formation were observed. Upon SEM/EDX analysis of the bone-implant interface, gaps that harbored layers containing carbon and oxygen were observed in the TiA group at 2 weeks. Fibrous tissue was detected in the space between the bone and the implant consistent with this layer. Alternatively, the observed carbon and oxygen may stem from the embedding medium. As the gap between the bone and the implant decreased, the amount of fibrous tissue decreased, which enhanced contact with the implant surface. Therefore, MgA does not cause serious local or systemic biological damage.

Magnesium alloys containing aluminum may have a latent toxic effects upon the human body. There is a report that no adverse effects of aluminum were observed in a study using Mg alloys with higher Al contents (9%) [20]. In the future, it is necessary to perform a cell viability test to confirm the safety of magnesium alloys containing aluminum for clinical applications.

### 4.5. Bone-Implant Interfacial Strength

The BIC by micro-CT analysis is only a measurement, but not a biomechanical parameter. In this study, biomechanical push-out testing was performed as one of the biomechanical parameters. MgA implants exhibited a significantly higher shear strength than TiA implants. A prior work investigated the interfacial shear strength of Mg alloys using the same method [32].

Castellani et al. reported that the differences in biomechanical parameters between Mg and Ti alloys were more pronounced when mechanical push-out testing was performed rather than micro-CT (e.g., at 4, 12 and 24 weeks after implantation, the maximum push-out force was 2.09-, 1.51- and 4.14-fold higher, and the BIC was 1.56-, 1.40- and 2.00-fold higher, respectively). The shear strength observed in the MgA group at 2, 4 and 12 weeks after implantation was 4.50-, 1.33- and 1.44-fold higher than values observed in the TiA group, respectively. Additionally, BV/TV values were 0.88-, 0.94- and 0.95-fold higher, and BIC values were 1.01-, 0.94- and 0.95-fold higher. As reported by Castellani et al., biomechanical methods enable a more sensitive evaluation of the bone-implant interface than micro-CT. However, there may be other reasons for the increased shear strength of the interface in the MgA group despite its lower BV/TV and BIC.

The high shear strength observed in the MgA and Mg groups is thought to be due to the increased surface roughness of the implant, which is caused by the corrosion of the Mg surface. Increasing the surface roughness of implants enhances their mechanical connection to the surrounding bone [47,48,49]. Shalabi et al. reviewed the effects of implant surface roughness in animals [50]. An increased surface roughness enhances BIC and increases interfacial shear strength. Therefore, the high shear strength of Mg-based materials may be due to the corrosion-induced increase in surface roughness.

The method used to determine the BIC area (*BIC*_Cg_) is a limitation of this study. We considered measuring the BIC using micro-CT to calculate shear strength. However, the BIC value represents the surface area of bone in the ROI around the implant, not the actual BIC area. Therefore, although the BIC value is suitable for evaluating osseointegration, it cannot be used to calculate shear strength. Therefore, we measured shear strength using a caliper gauge and calculated the contact area; this is a frequently used method [51,52,53]. Although this method did not take into account the difference in implant contact area (i.e., the implant surface area actually in contact with the bone) between the TiA group and the MgA, Mg and PLA groups, it is the most accurate method of measuring the contact area for shear strength calculation. In addition, when clinical use is assumed, an increase in the contact area (surface roughness) of the absorbable material is inevitable, which enhances the mechanical interlocking force with the bone. The shear strength determined in this study is considered clinically relevant, as it considers the influence of the interlocking force caused by the increase in surface roughness.

The reason as to why the ROI for the evaluation of BIC was defined as a one-voxel-thick ring, that was two voxels from the implant surface, was to avoid metal artifacts. When a substance to be scanned includes a substance having a significantly different X-ray transmittance, such as a metal and a bone, there is a problem that image noise is generated near a substance having a low transmittance. This noise is called artifact or metal artifact, because it is mainly caused by metal. If ROI is set directly on the implant surface, it gives a big estimate for BIC at the least due to metal artifacts.

## 5. Conclusions

The following results were obtained within the scope of this study. Mechanical push-out testing showed that MgA can obtain significantly higher bone-implant interface strength than TiA. Micro-CT analysis revealed no significant difference between TiA and MgA in BV/TV and BIC. Furthermore, it showed that MgA was superior to TiA in bone response. In this animal experiment, no serious inflammatory reaction, and no harm to the living body, were observed.

Our findings suggest that AZ31 MgA is suitable for MSIs and BFPs. However, most of the implant samples remained in situ after the 12-week experimental period, and so we could not reach conclusions regarding the entire degradation process. Therefore, longer-term studies are needed.

## Figures and Tables

**Table 1:** Composition of the metal samples as determined by X-ray fluorescence analysis (mass %).

Sample	Mg	Al	Ti	V	Mn	Fe	Cu	Zn
TiA		6.22	89.65	4.13				
MgA	97.96	1.08			0.33		0.01	0.62
Mg	99.97					0.03		

**Table 2:** Composition of 1000 mL of simulated body fluid (SBF).

Composition	Amount
NaCl	8.04 g
NaHCO_3_	0.36 g
KCl	0.23 g
K_2_HPO_4_·3H_2_O	0.23 g
MgCl_2_·6H_2_O	0.31 g
1.0 M HCl	39.00 mL
CaCl_2_	0.29 g
Na_2_SO_4_	0.07 g
Tris	6.12 g

**Table 3:** Threshold used for the extraction of implant samples and bone tissue (for 16-bit images).

Extraction Area	Extraction Level	Extraction Width
TiA	63,660	6000
MgA	45,015	4398
Mg	44,883	3725
PLA	37,464	5259
Cortical bone	48,000	Maximum
Trabecular bone	41,000	Maximum
